# Repurposing ARBs as treatments for breast cancer

**DOI:** 10.18632/aging.101249

**Published:** 2017-05-31

**Authors:** Amee J. George, Andrew Allen, Ashwini L. Chand

**Affiliations:** Cancer and Inflammation Laboratory, Olivia Newton-John Cancer Research Institute, Heidelberg, VIC, Australia

**Keywords:** angiotensin receptor blockers, breast cancer, cardiovascular disease, mammary tumour models

An increased understanding of G-protein-coupled receptors (GPCRs) has greatly influenced modern medicine, where approximately 30 to 50% of all marketed drugs act by binding to GPCRs. The angiotensin II receptor type 1 (AT_1_R), a classic seven transmembrane domain GPCR, is activated by the endogenously expressed peptide angiotensin II (AngII). Specific AT_1_R antagonists (angiotensin receptor blockers, ARBs) were first developed in the 1980s and are well characterised for their effects in treating cardiovascular disease (CVD). Extensive clinical studies indicating excellent patient tolerance to ARB treatment in the context of CVD, combined with recent evidence demonstrating a role for AT_1_R signalling in the pathogenesis of some forms of cancer, highlights the need to evaluate the therapeutic potential of re-purposing this class of medications in the treatment of cancer [1–3].

The Renin-Angiotensin System (RAS) is an endocrine and tissue-based regulator of cardiovascular, renal and neuroendocrine function. Hyper-activation of the AT_1_R leads to chronic inflammation associated with hypertension and cardiovascular disease. RAS components, including the AT_1_R, are expressed in normal tissue and cancers of the ovary, prostate, pancreas, breast and gut [4]. AT_1_R activation stimulates multiple signalling cascades critical for angiogenesis, vascular remodelling, cell proliferation, inflammation and fibrosis [4, 5]. Given the importance of these processes in cancer, inhibition of the AT_1_R provides a potential therapeutic target for treatment of solid malignancies. This hypothesis is strongly supported by the observed influence of AT_1_R blockade on cancer survival [1, 2, 6].

In a recent article, we present evidence that the therapeutic administration of the clinically approved ARB, *Losartan*, results in a significant reduction in tumour burden in a spontaneous mammary tumour model, and completely prevented tumour formation in 20% of treated mice [3]. This is the first *in vivo* demonstration that AT_1_R inhibition is a viable therapeutic option for breast cancer treatment. The cellular mechanisms via which the anti-tumourigenic effects were mediated included significant reduction in the production of the tumour inflammatory cytokines, TNFα and interleukin-6 [3]. This was accompanied by changes in the cellular transformation of tumour epithelial and stromal cells, resulting in tumours that had less invasive features [3]. In clinical biopsy samples, high AT_1_R expression positively correlated with ER-positive, HER2-negative, luminal A and B invasive ductal carcinomas [1–3].

The accumulation of functional data in experimental models of cancer and metastatic dissemination of cancer cells has now occurred over several decades. Effects of ARBs, including *Losartan, Candesartan, and Telmisartan* are also reported in the tumour-associated stroma, implying that these drugs may have dual effects on the tumour epithelial cells and the tumour microenvironment. As ARBs are *well tolerated*, orally administered, and off-patent (and therefore inexpensive), they make an appealing drug class for repurposing as cancer therapeutics. However, surprisingly there is little progress made in advancing this idea with assessments in cancer patients.

A potential clinical advantage for ARB use would be in targeting a specific risk for breast cancer recurrence and the management of CVD in postmenopausal women. Approximately 75% of all breast cancer cases occur in postmenopausal women; a cohort most at risk for CVD in females. Additionally, breast cancer survivors are at greater risk for CVD-related mortality compared with women without breast cancer and this increase in risk is manifested ~7 years after diagnosis. It accounts for ~35% of non-breast cancer mortality for survivors that are >50 years of age. Mortality attributed to CVD in breast cancer patients was increased in those who received chemotherapy [7]. In premenopausal women receiving adjuvant chemotherapy, which is associated with early menopause and an impact in CVD risk, a positive association with CVD was observed in patients with chemotherapy [7]. Given that CVD is a condition that emerges following long-term breast cancer therapy, it may be clinically warranted to assess whether CVD risks are reduced with ARB use in breast cancer patients undergoing chemotherapy.

To this effect, clinical investigations are required to determine the therapeutic efficacy of ARBs in the different breast cancer subtypes and treatment-resistant conditions; their combinatorial effects with current breast cancer therapeutics and whether co-morbidities such as CVD can be treated with the ARBs incorporated in breast cancer patient care. This represents an exciting opportunity to repurpose ARBs to improve patient outcomes, particularly in the context of breast cancer.
